# The Antimicrobial Domains of Wheat Puroindolines Are Cell-Penetrating Peptides with Possible Intracellular Mechanisms of Action

**DOI:** 10.1371/journal.pone.0075488

**Published:** 2013-10-02

**Authors:** Rebecca L. Alfred, Enzo A. Palombo, Joseph F. Panozzo, Mrinal Bhave

**Affiliations:** 1 Environment and Biotechnology Centre, Faculty of Life and Social Sciences, Swinburne University of Technology, Melbourne, Victoria, Australia; 2 Department of Environment and Primary Industries, Horsham, Victoria, Australia; Ghent University, Belgium

## Abstract

The puroindoline proteins (PINA and PINB) of wheat display lipid-binding properties which affect the grain texture, a critical parameter for wheat quality. Interestingly, the same proteins also display antibacterial and antifungal properties, attributed mainly to their Tryptophan-rich domain (TRD). Synthetic peptides based on this domain also display selectivity towards bacterial and fungal cells and do not cause haemolysis of mammalian cells. However, the mechanisms of these activities are unclear, thus limiting our understanding of the *in vivo* roles of PINs and development of novel applications. This study investigated the mechanisms of antimicrobial activities of synthetic peptides based on the TRD of the PINA and PINB proteins. Calcein dye leakage tests and transmission electron microscopy showed that the peptides PuroA, Pina-M and Pina-W→F selectively permeabilised the large unilamellar vesicles (LUVs) made with negatively charged phospholipids mimicking bacterial membranes, but were ineffective against LUVs made with zwitterionic phospholipids mimicking eukaryotic membranes. Propidium iodide fluorescence tests of yeast (*Saccharomyces cerevisiae*) cells showed the peptides were able to cause loss of membrane integrity, PuroA and Pina-M being more efficient. Scanning electron micrographs of PINA-based peptide treated yeast cells showed the formation of pits or pores in cell membranes and release of cellular contents. Gel retardation assays indicated the peptides were able to bind to DNA *in vitro*, and the induction of filamental growth of *E. coli* cells indicated *in vivo* inhibition of DNA synthesis. Together, the results strongly suggest that the PIN-based peptides exert their antimicrobial effects by pore formation in the cell membrane, likely by a carpet-like mechanism, followed by intracellular mechanisms of activity.

## Introduction

The puroindoline (PIN) proteins of wheat are unique in that, on the one hand, they determine one of the commercially most important characteristics of wheat, i.e., whether the grain texture is soft or hard, and on the other, they also exhibit the ability to kill bacterial and fungal cells. While seemingly unrelated, both properties appear to hinge on the unique biochemical properties of these proteins. PINA and PINB are small (pre-proteins: 148 amino acids; mature proteins: 119–120 amino acids), highly basic (pI 10.5), lipid-binding proteins. The proteins have ten highly conserved Cys residues, eight of which form a specific pattern known as the ‘eight-cysteine motif (8CM)’ [1], a tertiary structure of four α-helices held by five disulphide bonds, and a unique domain called the ‘tryptophan-rich domain’ (TRD). The TRD is composed of five Trp residues in PINA or three in PINB, interspersed with the basic residues Arg and/or Lys [2], [3]. The dominant ‘soft’ grain texture of wheat (suitable for products such as cakes and cookies) requires both PINA and PINB to be present in their ‘wild-type’ form, and the lack of, or amino acid substitutions in, either PIN protein result in hard grain textures (suitable for products such as breads) [4]. The presence/absence of the PIN proteins in the wheat grain significantly influences the milling behaviour, mill settings, flour properties, as well as the quality and properties of the end-use products [5]. The *Pin* genes and the various ‘hardness’ alleles have been reviewed in Bhave and Morris [6].

Since their discovery, the PIN proteins have been suggested to be membranotoxins, with roles in seed or seedling defence against microbial pathogens [2]. The association of PINs with the starch granule surface (imparting the effects on grain texture) [6], [7], the suggested *in vivo* defence roles, and observed *in vitro* antimicrobial properties all appear to be related to their tertiary structure and lipid-binding nature [8]. The *in vivo* defence roles in wheat seed are as yet unproven; however, the purified or expressed PINA and PINB proteins exhibit various degrees of antimicrobial activity against several Gram-positive and Gram-negative bacteria and/or fungi [9]–[11], including *Staphylococcus epidermidis* that causes skin infections [12]. There is also strong evidence from transgenic plant work that they indeed causatively impart antifungal defence to the host plant [13]–[15] and *in vivo* seed defence [16]. Synthetic peptides mimicking the TRDs of PINA and PINB also exhibit significant activity against both Gram-positive and Gram-negative bacteria [17]. We found that a number of synthetic peptides based on the TRDs of the wild-type and mutant PINs as well as the related barley hordoindolines were variously active against bacteria and/or phytopathogenic fungi [18]. The antimicrobial activity was found to be associated with the TRD, and certain substitutions within it affected this activity at both quantitative (in terms of the minimum inhibitory concentration (MIC) of a peptide against an organism) and/or qualitative (in terms of susceptible species) levels. We have also shown the peptides to be effective against the rust diseases of wheat, which are pathogens of global concern [19].

The PIN-based peptides are a class of antimicrobial peptides (AMPs) called the cationic antimicrobial peptides (CAPs) [20] due to their net positive charge, and are also called Trp-rich AMPs, due to their TRD. While the reported natural and synthetic Trp-rich CAPs have some sequence variations and display a range of antibacterial, antifungal and/or antiviral activities, and some also antitumor activities, they are highly conserved in the nature of the first step of their activity, i.e., initial interaction with the target membrane. Due to the positively charged side chains of CAPs and the negatively charged components such as the phosphate groups in the lipopolysaccharides of Gram-negative bacteria and lipoteichoic acids of Gram-positive bacteria, it is widely accepted that this interaction is electrostatic and not receptor mediated [21]. After the attachment, several models have been proposed for the mechanisms by which CAPs permeabilise cell membranes [21], [22]. AMPs can be further divided into those that cause cell death by direct cell lysis, and those that disrupt membranes without lysis, to affect critical intracellular targets such as DNA, RNA, protein or cell wall syntheses processes, or other enzymatic activities, thus killing the cell [21]. The Trp residues in the TRD and the basic nature of the PIN proteins/peptides are proposed to be important for their preferential binding to negatively charged lipids compared to neutral ones, and thus their greater antimicrobial activity but little haemolytic activity on mammalian cells [17], [23]. The Arg residue adjacent to a Trp in the TRD of PINA (but not PINB) may also enhance the membrane insertion of Trp [17]. The mechanism of activity of PIN proteins and/or peptides may be membrane leakiness caused by perturbation of lipid packing [17], [24], rather than via protein channels or pores. The peptides appear too small to form membrane-spanning pores; however, the reports of cation channels formed by PINA [25], [26] suggest the structural properties of the larger protein may enable channel formation. The present work investigates the mechanisms of activity of the PIN-based peptides against microbial cells, through a number of independent techniques. The results have implications to understanding the roles of PINs in influencing grain hardness and any *in vivo* roles in defence of the seed/plant from pathogens, as well as emerging applications of PIN-based synthetic peptides as antimicrobial peptides.

## Materials and Methods

### Design of Peptides

Custom peptides PuroA and PuroB were modelled on the TRD of the PINs encoded by the wild-type alleles *Pina-D1a* and *Pinb-D1b,* respectively [2]. The peptide designated Pina-W→F was designed with all Trps substituted with Phe residues, and Pina-R39G had the Arg-39 substituted for Gly-39. Pina-M, Pinb-B, Pinb-D, Pinb-L and Pinb-Q were based on the TRD of the natural hard grain associated alleles *Pina-D1m* [27], *Pinb-D1b* [28], *Pinb-D1d* [29], *Pinb-D1l* [30] and *Pinb-D1q* [31], respectively (Table 1). All peptides were amidated at the C-terminus (NH_2_). The peptides were synthesised at >95% purity by Biomatik Corp (Ontario, CA) by solid-phase methods using N-(9-fluorenyl) methoxycarbonyl (Fmoc) chemistry. Peptide solutions prepared as detailed previously [18].

**Table 1:** Peptides investigated in this study.

Peptide	Gene encoding this TRD;GenBank accession/Reference	Peptide sequence^a^	Net charge, numberof Trp residues	pI^b^	MW^c^
Indolicidin	Selsted et al. [44]	ILPWKWPWWPWRR-NH_2_	+3; 5	12.01	1809.22
PuroA	Pina-D1a; DQ363911	**FPVTWRWWKWWKG-NH_2_**	+3; 5	11.17	1862.23
Pina-M	Pina-D1m; EF620907	F***S***VTWRWWKWWKG-NH_2_	+3; 5	11.17	1852.19
Pina-R39G	Earlier work [18]	FPVTW***G***WWKWWKG-NH_2_	+2; 5	10.00	1667.05
Pina-W→F	Earlier work [18]	FPVT***F***R***FF***K***FF***KG-NH_2_	+3; 0	11.17	1763.10
PuroB	Pinb-D1a; DQ363913	**FPVTWPTKWWKG-NH_2_**	+2; 3	10.00	1531.84
Pinb-B	Pinb-D1b; DQ363914	FPVTWPTKWWK***S***-NH_2_	+2; 3	10.00	1561.86
Pinb-D	Pinb-D1d; Lillemo and Morris [29]	FPVTWPTKW***R***KG-NH_2_	+3; 2	11.17	1501.81
Pinb-L	Pinb-D1l; Pan et al. [30]	FPVTWPTKWW***E***G-NH_2_	0; 3	6.00	1532.78
Pinb-Q	Pinb-D1q; EF620909	FPVTWPTKW***L***KG-NH_2_	+2; 2	10.00	1458.78
GSP-5D	Gsp-1-5D; CR626934	MPLSWFFPRTWGKR-NH_2_	+3; 2	12.01	1808.20
Hina	Hina; AY644140	FPVTWRWWTWWKG-NH_2_	+2; 5	11.00	1836.15
Hinb1	Hinb-1; AJ276143	FPLTWPTKWWKG-NH_2_	+2; 3	10.00	1545.86
Hinb1a	Hinb-1; AY644058	FPLTCPTKWWKG-NH_2_	+2; 2	9.31	1462.79

a Peptides based on the TRD sequences of the wild-type PINA and PINB proteins are shown in bold. Amino acid substitutions in relation to these are shown in bold italics;

b predicted using the Compute pI/MW Tool’ at ExPAsy (http://au.expasy.org/tools/pi_tool.html);

c molecular weights were determined by mass spectrometry (Biomatik, USA).

### Preparation of Large Unilamellar Vesicles (LUVs)

A calcein leakage assay as previously described [17] was performed to determine the effects of PIN-based peptides on the permeability of synthetic large unilamellar vesicles (LUVs), designed to mimic bacterial and mammalian cell membranes. The phospholipids used were DOPC (1,2-dioleoyl-sn-glycero-3-phosphocholine), with zwitterionic head groups and DOPG (1,2-dioleoyl-sn-glycerol-3-[phosphor-rac-(1-glycerol)]), with negatively charged headgroups (Avanti® Polar lipids, Alabaster, USA). These were used to make DOPC:DOPG (1∶3) LUVs to mimic bacterial cell membranes, or DOPC only, to mimic mammalian cell membranes [17]. Calcein-entrapped LUVs were prepared by the extrusion method using an Avanti mini extrusion apparatus (Avanti® Polar Lipids, Alabaster, USA). The DOPC:DOPG (a 1∶3 mixture) or DOPC were dissolved in chloroform (25 mg/mL) and aliquoted into glass test tubes to give a concentration of 2.6 mM when resuspended in 2 mL buffer. Chloroform was evaporated under a stream of N_2_ and the lipid films were dried overnight under vacuum. The films were then resuspended in 2.0 mL of 70 mM calcein buffer (in 10 mM Tris-HCl, pH 7.4) and incubated at room temperature for 30 minutes. To increase entrapment of the dye, five repeated freeze\thaw cycles were used by placing the vial in liquid N_2_ and then a warm water bath (37°C). The lipid mixture was then extruded through the mini-extruder 10 times through two stacked 0.1 µm pore-size filters. Free calcein was removed by centrifugation (10, 000×*g*, 10 min) three times and washing with 10 mM Tris, 100 mM NaCl buffer (pH 7.4) [32]. Standards of phosphorus (inorganic P; Pi) were prepared (0.05–1.5 mg/mL) using KH_2_PO_4_ and read at 820 nm and a linear standard curve was generated (Figure S1 in File S1). The phospholipid concentration of the vesicle preparations was determined by a total phosphorus assay [33]. The assay reagent was prepared fresh by mixing 1 part of 1% ascorbic acid with 6 parts of 0.42% ammonium molybdate•4H_2_O in H_2_SO_4_ and stored on ice. 7 µL of it was added to 3 µL of vesicle suspensions and incubated for 20 min at 45°C. The absorbance of the samples was noted at 820 nm, the Pi concentration of each was calculated from the Pi standard curve, and this value multiplied by a factor of 25 to convert Pi to phospholipid concentration [34]. The standard curve of phosphorus standards (Figure S1 in File S1) was used to determine the Pi concentration in a LUV preparation, and this was converted to phospholipid concentration (Table S1 in File S1) to adjust the preparations to the required 10 µM phospholipid concentration prior to peptide treatment [17].

### Dye Leakage Experiments

The calcein-filled LUVs were diluted with 10 mM Tris, 100 mM NaCl buffer (pH 7.4) to a final phospholipid concentration of 10 µM. The experiments were performed in a Varian Cary Eclipse fluorescence spectrophotometer with excitation and emission wavelengths of 496 nm and 515 nm, slit widths of 20 mM and a PMT detector of 600 V. The peptides (PuroA, Pina-M, Pina-W→F and PuroB) were added to LUVs to a final peptide concentration of 8–125 µg/mL in 100 µL volumes and the fluorescence (F) monitored for 5 min at 60 s intervals. The total calcein fluorescence (F_T_) was determined by addition of 1.0% Triton X100. The dye leakage was calculated as % Leakage = ((F – F_0_)/(F_T_ – F_0_)), F_0_ being fluorescence of each sample at T = 0.

### Transmission Electron Microscopy (TEM) of LUVs

Suspensions of untreated and peptide-treated DOPC or DOPC:DOPG (1∶3) LUVs (prepared as above with peptide concentrations of the respective MICs for *Escherichia coli*) were pipetted onto the surface of Formvar coated copper grids (ProSciTech, Thuringowa, Australia) and washed twice with distilled H_2_O. The grids were then stained with 0.5% uranyl acetate for approximately 2 seconds [35] and the excess stain removed by blotting on Whatman paper. The grids were then air-dried and inspected at La Trobe University with either a JEOL JEM-2010HC or a JEM-120ex STEM/TEM transmission electron microscope (Tokyo, Japan).

### Minimum Inhibitory Concentrations (MIC) of the Peptides against Yeast Cells

*Saccharomyces cerevisiae* (ATCC 287) was grown in Potato Dextrose Broth (PDB) overnight at 30°C with shaking at 220 rpm [36]. The cultures were vortexed vigorously and the cell suspension adjusted to 1–5×10^6^ cells/mL with PDB (McFarland standard 0.5) and then diluted 1∶200 with PDB to a final concentration of 0.5–2×10^3^ cells/mL [37]. The MIC assays were carried out in 96-well plates as described previously [18]. Peptides (PuroA, Pina-M, Pina-W→F and PuroB) in 25 µL volumes were added to the first empty well and a two-fold dilution was carried out across each row with a starting peptide concentration of 250 µg/mL and a final one of 2 µg/mL. Test wells were subsequently inoculated with 75 µL yeast cells (0.5–2×10^3^ cells/mL) and incubated overnight at 30°C. Negative control (no peptide) and positive control (fungicide Mycobutanil) wells were also prepared. The MIC was defined as the lowest concentration of the peptide required to inhibit fungal growth [37], observed visually.

### Propidium Iodide uptake by Yeast Cells

*S. cerevisiae* was grown overnight at 30°C in 100 mL PDB and the cells collected by centrifugation and resuspended in Phosphate Buffered Saline (PBS: 137 mM NaCl, 2.7 mM KCl, 8 mM Na_2_HPO_4_, 1.46 mM KH_2_PO_4_; pH 7.4) to ∼1.5×10^7^ cells/mL (OD_600_ = 0.6–0.8) [36]. 75 µL of the suspension was incubated with 25 µL of stock solutions of PuroA, Pina-M, Pina-W→F, PuroB and indolicidin (to give a final peptide concentration of 64, 125, 250 or 500 µg/mL), or with PBS (for no-peptide controls) for 1 h at 30°C, followed by staining with propidium iodide (PI) as described [38]. PI stock solution (60 µg/mL) was prepared in MilliQ water and stored in dark at −20°C. It was diluted to 12 µg/mL before use, mixed 1∶1 with peptide-treated or untreated yeast cells (to final PI concentration 6 µg/mL), and the samples incubated in the dark for 5 min. A 10 µL aliquot of these was examined using a Nikon Eclipse 50 i fluorescence microscope with 561 nm excitation and 630/22 emission filter, at 400×magnification. The photomicrographs were taken under light and fluorescence for each field. Five randomly selected fields were recorded and scored for each sample.

### Scanning Electron Microscopy (SEM) of Peptide-treated Yeast Cells

*S. cerevisiae* cultures were grown overnight in 5.0 mL of PDB 30°C, to OD_600_ = 1.2–1.8 (∼5×10^7^ cells/mL) [36]. 75 µL aliquots of the yeast cell suspension were incubated with 25 µL of PuroA, Pina-M, Pina-W→F or PuroB (at final concentrations of 0.5×, 1× or 2× of the respective MIC for *S. cerevisiae;* see results) for 1 h at 30°C. The cells were collected by centrifugation (1000×*g*, 5 min), the pellets washed three times in PBS (pH 7.4) and resuspended in 100 µL MilliQ (ultra-pure) water. A 25 µL aliquot of the cell suspension was spotted onto a glass slide, air-dried, then fixed and dehydrated as described previously [39]. In brief, the air-dried slides were fixed in 2.5% glutaraldehyde (in PBS, pH 7.4) overnight in a humid chamber, then washed with PBS pH 7.4 for 10 min, and dehydrated in an ethanol gradient of 50%, 60%, 70%, 80%, 90% and 100%. The slides were freeze-dried overnight and then coated in a Dynavac CS300 coating unit with carbon and gold, with double sided conducting carbon tape attached to the slides for better conductivity. The samples were analysed using a ZEISS supra 10 VP field emission scanning electron microscope (Carl Zeiss Microscopy, NY, USA) at 3.0 kV.

### Gel Retardation Assay for Testing *in vitro* DNA-binding Ability of Peptides

This test assesses any peptide-DNA binding by noting the retardation of the rate of migration of DNA bands through agarose gels. A number of methods were tested. As per Hsu et al. [40], DNA of a plasmid (200 ng) or a commercial molecular weight marker (50 ng) was mixed with a peptide (to final concentrations of 32, 64, 125 or 250 µg/mL) in 15 µL of 10 mM Tris-1 mM EDTA buffer (pH 8.0), incubated at room temperature for 2 min, then electrophoresed in 0.5% or 1.0% agarose gels containing 0.5 µg/mL ethidium bromide (EtBr). Following Park et al. [41], 100 ng plasmid DNA was mixed with a peptide (to different concentrations) in 20 µL binding buffer (5% glycerol, 10 mM Tris-HCl (pH 8.0), 1 mM EDTA, 1 mM DTT, 20 mM KCl, 50 µg/mL BSA), held for 1 h at room temperature, then electrophoresed in EtBr-containing gels. Finally the protocols were modified to eliminate any interference of EtBr in the initial peptide-DNA binding. Plasmid DNA (100 ng) was mixed with a peptide (to final concentrations of 16, 32, 64, 125, 250 or 500 µg/mL) in 10 µL of MilliQ water, held at room temperature for 1 h and electrophoresed, followed by emersion of gels in EtBr solution (0.5 µg/mL) for 30 min before imaging.

### *E. coli* Filamentation Assay for Testing *in vivo* Inhibition of DNA Synthesis by Peptides

The method as described [42] was used to assess if the PIN-based peptides cause filamentation of *E. coli* cells, indicative of inhibition of *in vivo* DNA synthesis. *E. coli* (ATCC 25922) grown to logarithmic phase (OD_600_ = 0.2) was diluted to OD_600_ of ∼0.04. 75 µL of this culture was mixed with 25 µL of a peptide solution (to a final peptide concentration of 2–250 µg/mL) and incubated for 3 h at 37°C. 50 µL of the sample was spotted onto a glass slide, air dried, and stained with Crystal Violet for 1 min. Peptide mixed with water was used as negative control, and the Trp-rich AMP indolicidin (ILPWKWPWWPWRR-NH_2_) was used as a positive control due to its known filamentation effect [42]. All samples were tested in triplicate. The cells were observed by light microscopy (1000×magnification).

## Results

### Selective Permeabilisation of Negatively Charged Vesicles by PINA-based Peptides

The peptides PuroA (FPVTWRWWKWWKG-NH_2_) (designed based on the TRD of the wild-type PINA), Pina-M (FSVTWRWWKWWKG-NH_2_) (designed based on a natural hard wheat mutant), and Pina-W→F (FPVTFRFFKFFKG-NH_2_) (with all Trps substituted with Phe), were found to exhibit the strongest inhibitory activities against bacteria and phytopathogenic fungi amongst a number of peptides tested earlier [18]. The present work investigated whether these activities were due to membrane disruption, by studying the release of the fluorescent dye calcein from the LUVs made using a mixture (1∶3) of DOPC (zwitterionic) and DOPG (negatively charged) phospholipids to mimic bacterial membranes, or DOPC only, to mimic mammalian membranes [17]. The phospholipid concentration of the extruded calcein-filled LUVs was calculated using a total phosphorus assay [33]. The no-peptide control LUVs in the PuroA experiments showed a background fluorescence of 28% (for DOPC:DOPG LUVs) and 30% (for DOPC LUVs), probably due to the free calcein and/or spontaneously lysed vesicles. PuroA induced 70–90% dye leakage from the DOPC:DOPG LUVs at 5 min incubation when used at concentrations equivalent to, or higher than, its MIC against *E. coli* cells (16 µg/mL) established earlier [18] (Table 2; Figure S2 in File S1). None of the tested concentrations were able to induce 100% leakage (i.e., complete lysis). When tested with the DOPC LUVs, PuroA (16 µg/mL) showed no additional impact as compared to the no-peptide negative control (30% leakage), and slightly higher leakage (42%) at the highest concentration tested (Figure S2 in File S1). The no-peptide controls of Pina-M assays showed a similar background fluorescence (28%, DOPC:DOPG; 26%, DOPC). Pina-M showed results similar to PuroA, i.e., 75% leakage when used at its MIC against *E. coli* (13 µg/mL) and a maximum of 85% with the DOPC:DOPG LUVs. With the DOPC LUVs, Pina-M had results similar to the control, and slightly higher % leakage at the highest concentration tested (Figure S2 in File S1). The non-antibacterial peptide PuroB showed levels of dye leakage from the DOPC:DOPG (bacteria-mimicking) LUVs exceeding those of no-peptide control only at its highest concentration tested (250 µg/mL, i.e., 15 or 30 times more than PuroA or Pina-M, respectively) (Table 2; Figure S2 in File S1), and had negligible effects on the DOPC LUVs. Further, the Trp to Phe substitution peptide Pina-W→F induced less dye leakage (64%) from the DOPC:DOPG LUVs at its MIC against *E. coli* (32 µg/mL) compared to PuroA, and no significant leakage from the DOPC LUVs, even at high concentration (Table 2; Figure S2 in File S1). The results indicate that the peptides disrupted the negatively charged phospholipid vesicles but did not cause complete lysis under the conditions used, and were ineffective against zwitterionic lipid vesicles.

**Table 2:** Calcein dye leakage from large unilamellar vesicles treated with peptides.

Sample	% Calcein leakage fromDOPC:DOPG LUVs*	% Calcein leakage fromDOPC LUVs*
No-peptide (negative control)	28.5	28.0
Triton x100 (positive control)	99.0	99.0
PuroA 8 µg/mL	31.0	30.1
PuroA 16 µg/mL	71.5	33.0
PuroA 32 µg/mL	79.5	34.5
PuroA 64 µg/mL	75.1	38.5
PuroA 125 µg/mL	91.0	46.0
Pina-M 8 µg/mL	59.0	31.0
Pina-M 16 µg/mL	74.5	30.0
Pina-M 32 µg/mL	84.5	33.5
Pina-M 64 µg/mL	81.5	44.0
Pina-M 125 µg/mL	88.5	54.5
Pina-W→F 8 µg/mL	29.2	28.0
Pina-W→F 16 µg/mL	32.0	27.0
Pina-W→F 32 µg/mL	64.0	27.0
Pina-W→F 64 µg/mL	78.0	31.0
Pina-W→F 125 µg/mL	81.0	36.0
PuroB 16 µg/mL	28.0	30.0
PuroB 32 µg/mL	29.0	30.0
PuroB 64 µg/mL	29.0	33.0
PuroB 125 µg/mL	35.0	32.1
PuroB 250 µg/mL	56.0	31.0

* average of duplicate experiments; the variation between the duplicate values was in the range of 0.7 to 2.8% for all experiments.

**Figure 1:**
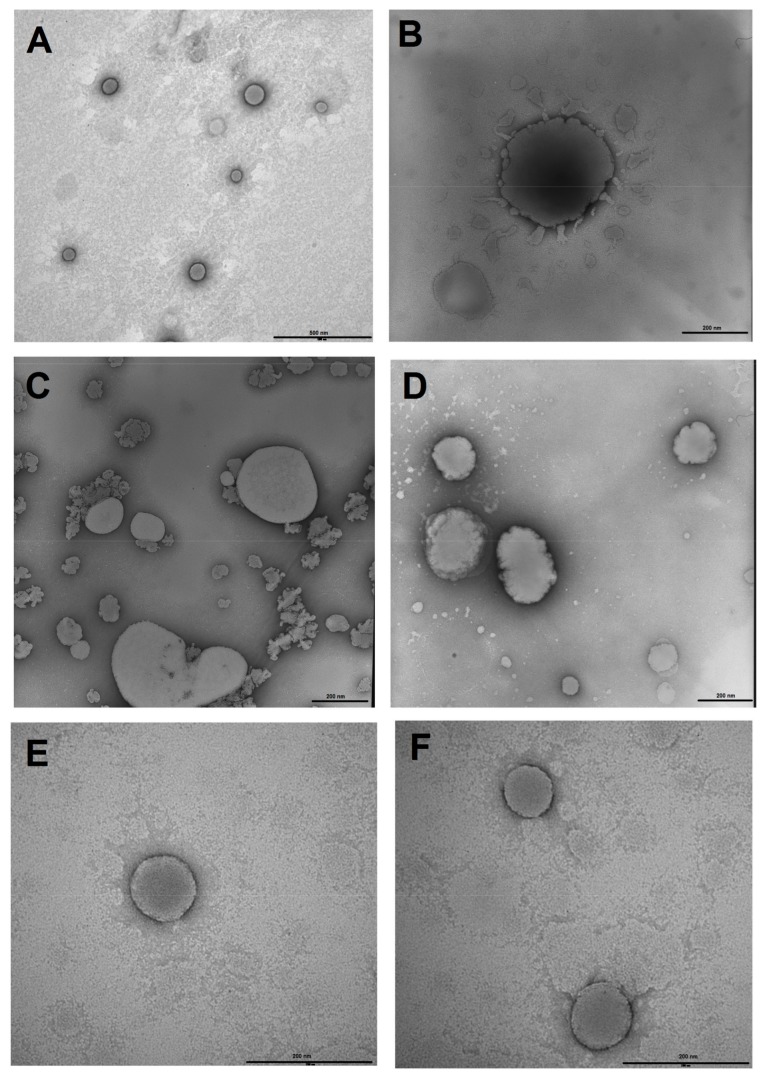
Transmission electron micrographs of negatively stained calcein-containing DOPC LUVs treated with peptides. **A.** Untreated DOPC:DOPG LUVs, magnification 36,000×, scale bar 500 nm; **B.** DOPC:DOPG LUVs treated with PuroA, magnification 40,000×, scale bar 200 nm; **C.** DOPC:DOPG LUVs treated with PuroA, magnification 25,000×, scale bar 200 nm; **D.** DOPC:DOPG LUVs treated with Pina-M, magnification 30,000×, scale bar 200 nm; **E.** Untreated DOPC LUVs, magnification 50,000×, scale bar 200 nm; **F.** DOPC LUVs treated with Pina-M, magnification 50,000×, scale bar 200 nm.

The results were further investigated by visualising the LUVs by TEM. The untreated LUVs ranged from 100–200 nm in diameter and appeared spherical, with a smooth, defined border (Figure 1A). Treatment of the DOPC:DOPG LUVs with PuroA (at 16 µg/mL; its MIC against *E. coli*) resulted in a number of morphological changes, e.g., the surface (edge) appearing scalloped with protrusions, suggesting a loss of integrity, leakage of contents, and 2–4 times larger sized (300–400 nm) (Figure 1B, C) also suggesting membrane permeation and diffusion of water into the vesicles. A survey of five random fields did not indicate a decrease in the number of LUVs in the treated sample (data not shown), suggesting that a majority of vesicles were not lysed after 5 min incubation. The DOPC:DOPG LUVs treated with Pina-M (at 13 µg/mL; its MIC against *E. coli*) also showed similar changes (Figure 1D). Treatment of the DOPC LUVs with PuroA or Pina-M resulted in no observable changes to vesicle morphology, as compared to the untreated LUVs (data not shown). The results were consistent with the calcein dye leakage findings.

### Permeabilisation of Membranes of Yeast Cells by PIN-based Peptides

The unicellular fungal cells (*S. cerevisiae*) are more readily visualised by light microscopy without oil immersion due to their larger size (10–12 µm), compared to the LUVs (100–200 nm) or bacterial cells (0.5–2 µm). *S. cerevisiae* membranes, like bacterial membranes, are highly electronegative due to phosphatidylserine lipids [22]. The peptides PuroA, Pina-M, Pina-W→F, PuroB (see above) and indolicidin were found to inhibit the growth of *S. cerevisiae* at MICs of 125 µg/mL, 125 µg/mL, 250 µg/mL, 250 µg/mL, and 125 µg/mL, respectively. The propidium iodide (PI) uptake assay was used to assess the effects of peptides on membrane integrity of yeast cells *in vivo*. PI fluoresces when bound to nucleic acids, but intact membranes are impermeable for it; hence it can be used to measure membrane permeability changes under various conditions [38]. The untreated control cultures showed a small number of fluorescent cells (5.7%), possibly due to dead cells that had become permeable (Figure S3 in File S1; Table 3). Incubation with PuroA or Pina-M showed PI uptake by 100% of cells at 64 µg/mL, i.e., below its MIC against yeast. The peptide Pina-W→F led to 56–72% of cells becoming permeable to the dye at sub-MIC levels, and 100% of the cells at MIC levels and higher. PuroB led to no observable effects at sub-MIC levels, moderate uptake (29.4% cells) at MIC, and 100% of the cells being fluorescent at higher concentrations (Figure S3 in File S1).

**Table 3:** Propidium iodide uptake by yeast cells treated with peptides.

Peptide*	% fluorescent cells**
No-peptide (negative control)	5.7
Indolicidin (positive control) 125 µg/mL	100
PuroA 64 µg/mL	100
PuroA 125 µg/mL	100
PuroA 250 µg/mL	100
PuroA 500 µg/mL	100
Pina-M 64 µg/mL	100
Pina-M 125 µg/mL	100
Pina-M 250 µg/mL	100
Pina-M 500 µg/mL	100
Pina-W→F 64 µg/mL	56.5
Pina-W→F 125 µg/mL	72.3
Pina-W→F 250 µg/mL	100
Pina-W→F 500 µg/mL	100
PuroB 64 µg/mL	6.8
PuroB 125 µg/mL	7.2
PuroB 250 µg/mL	29.4
PuroB 500 µg/mL	100

* MICs of PuroA, Pina-M, Pina-W→F and PuroB for *S. cerevisiae*: 125 µg/mL, 125 µg/mL, 250 µg/mL and 250 µg/mL, respectively.

** Average value of 4 fields counted at 400×magnification, from each of two independent experiments. The value of 100% indicates all cells were fluorescent in the duplicate experiments with these peptides. For experiments that showed <100% fluorescent cells, the variation between the duplicates was in the range of 0.9 to 5.2%.

### Induction of Pore Formation in Yeast Cell Membranes by PuroA

**Figure 2:**
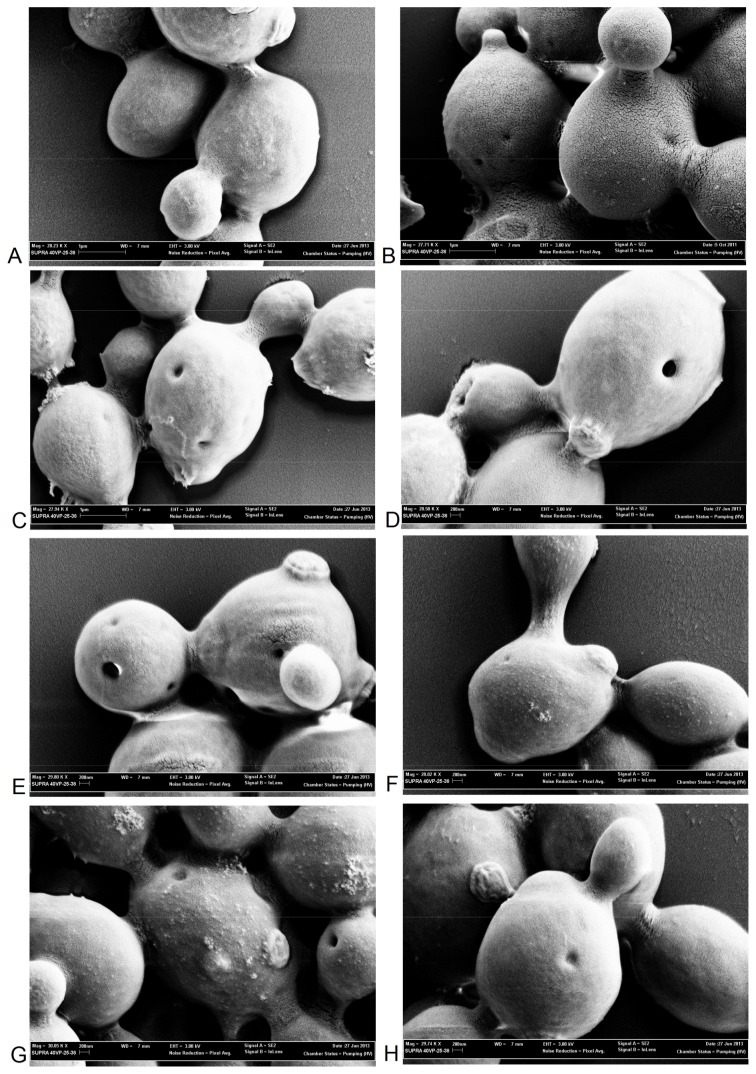
Scanning electronmicrographs of *S. cerevisiae* cells treated with PuroA, Pina-M, Pina-W→F and PuroB. **A.** No-peptide control, magnification 28,000×, scale bar 1 µm; **B.** PuroA 64 µg/mL, magnification 28,000×, scale bar 1 µm; **C.** PuroA 125 µg/mL, magnification 28,000×, scale bar 1 µm; **D.** Pina-M 64 µg/mL, magnification 28,000×, scale bar 200 nm; **E.** Pina-M 125 µg/mL, magnification 29,000×, scale bar 200 nm; **F.** Pina-W→W 125 µg/mL, magnification 28,000×, scale bar 200 nm; **G.** Pina-W→W 250 µg/mL, magnification 29,000×, scale bar 200 nm; **H.** PuroB 250 µg/mL, magnification 29,000×, scale bar 200 nm.

SEM was used to further investigate the effects of PuroA, Pina-M, Pina-W→F and PuroB on membranes of individual *S. cerevisiae* cells. At sub-MIC level (64 µg/mL and 125 µg/mL. respectively), the surface of cells treated with PuroA and Pina-W→F appeared smooth, similar to untreated cells, but interestingly, many of the treated cells exhibited shallow pits or pores on the surface (Figure 2B and 2F). At the MIC (125 µg/mL and 250 µg/mL, respectively) and higher concentrations, the pits were more pronounced and some cellular material appeared to be leaking through the membrane, forming protrusions (Figure 2C and 2G). Pina-M showed stronger membrane penetrating activity of all the peptides tested, with pronouced pits observed at sub-MIC level (64 µg/mL) and higher concentrations (Figure 2D and 2E). For PuroB, with a TRD truncated to only 2 Trps, many of the cells showed shallow pores and leakage of cellular material at MIC levels and below (Figure 2H), while at higher concentrations the pits appeared more pronounced.

### *In vitro* DNA-binding Ability of PIN-based Peptides

**Figure 3:**
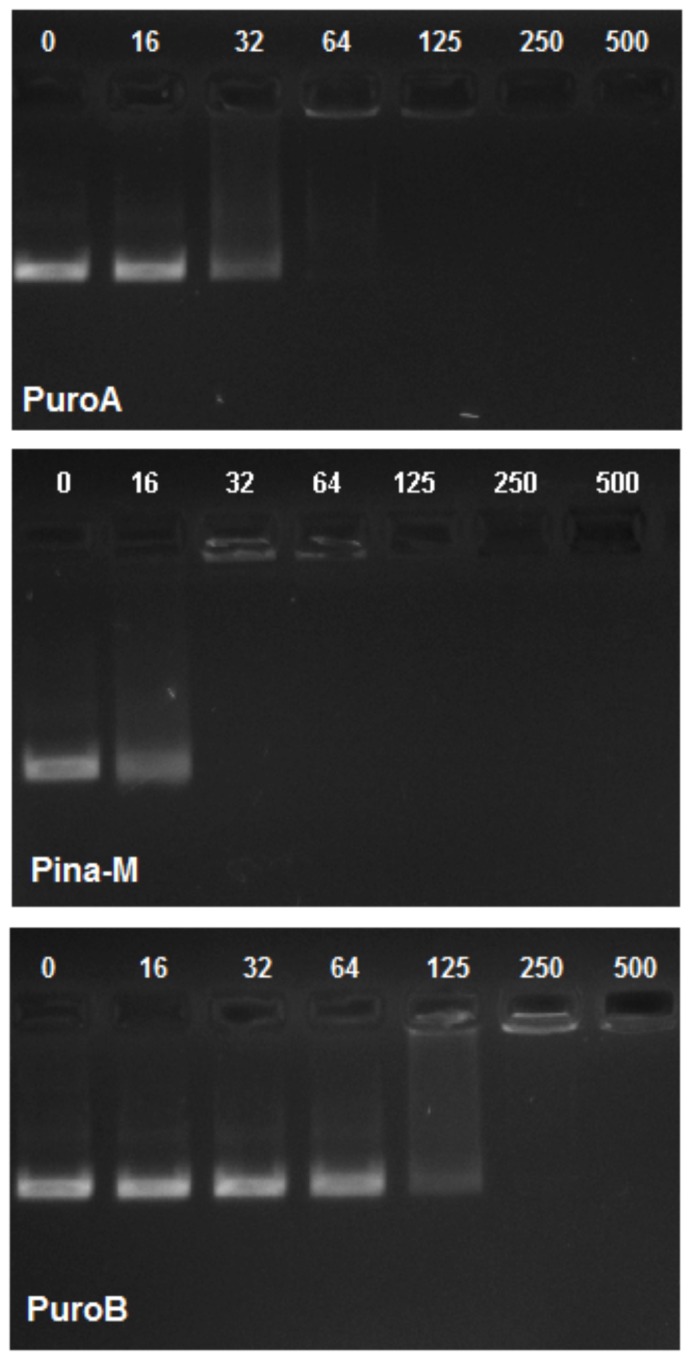
Interaction of peptides with plasmid DNA. Binding of peptides to DNA assessed by measuring the retardation of plasmid DNA (pBluescript SK+, 100 ng) migration through an agarose gel. The peptide concentration indicated above each lane represents 0, 16, 32, 64, 125, 250 and 500 µg/mL. A: PuroA; B: Pina-M, C: PuroB.

Inhibition of DNA synthesis is the mode of action of some AMPs that inhibit bacterial growth without causing cell lysis [41], [42]. Thus, in order to test whether PIN-based peptides (tested above) may use this mechanism (in addition to the cell leakage shown above); their *in vitro* DNA-binding ability was investigated by a gel retardation assay. Additionally, peptides based on the related barley hordoindolines (HIN) and the wheat Grain softness protein-1 (GSP-1) proteins, which had been previously found to have antimicrobial activities [18], were included. The methods described previously [40], [41] were unsuccessful in detecting any retardation of movement of peptide-bound DNAs (results not shown), possibly due to the EtBr in the gels interfering with access of peptides to DNA. Adoption of post-electrophoresis EtBr staining led to significant results. The peptides PuroA, Pina-M, Pina-R39G, Pina-W→F, HinA and GSP-5D, completely inhibited the migration of plasmid DNA through the gels at concentrations of 32 (Pina-M), 64 (PuroA, Pina-W→F, GSP-5D, Hina, indolicidin), or 125 (Pina-R39G) µg/mL, showing a strong DNA binding ability (Figure 3; Figure S4 in File S1; Table 4). These peptides also exhibit a range of antimicrobial activities, as shown earlier [41]. Of the PINB-based peptides, PuroB completely inhibited the DNA migration at a higher concentration (250 µg/mL), and all other Pinb and Hinb peptides showed only partial inhibition at 500 µg/mL. In general, a majority of peptides that showed the highest affinity for DNA (as seen by gel retardation) also had the highest predicted net charge (+3), while those that were not able to effect gel retardation generally had a net charge of 0 to +2 (Table 1).

**Table 4:** Gel retardation and *E. coli* cell filamentation assays for peptide-DNA binding.

Peptide	Peptide concentration (µg/mL) inducinggel retardation^a^		Minimum peptideconcentration (µg/mL) inducingfilamentation in*E. coli*	MIC (µg/mL) against*E. coli cells* ^b^
	Partial	Full		
Indolicidin	32±0	64±0	32±0	32±0
PuroA	32±0	64±0	16±0	16±0
Pina-M	16±0	32±0	8±0	13±5
Pina-R39G	64±0	125±0	64±0	64±0
Pina-W→F	32±0	64±0	nf	32±0
PuroB	125±0	250±0	250±0	>250
Pinb-B	>500	>500	nf	>250
Pinb-D	500±0	>500	nf	>250
Pinb-L	>500	>500	nf	>250
Pinb-Q	500±0	>500	nf	>250
GSP-5D	32±0	64±0	64±0	64±0
Hina	32±0	64±0	32±0	32±0
Hinb1	500±0	>500	250±0	>250
Hinb1a	500±0	>500	250±0	>250

a Values from triplicate assays; nf: no filamentation induced at the maximum final concentration of peptide tested, 250 µg/mL and ^b^data from previous work [18].

### Inhibition of DNA Synthesis *in vivo* in *E. coli* by PIN-based Peptides

**Figure 4:**
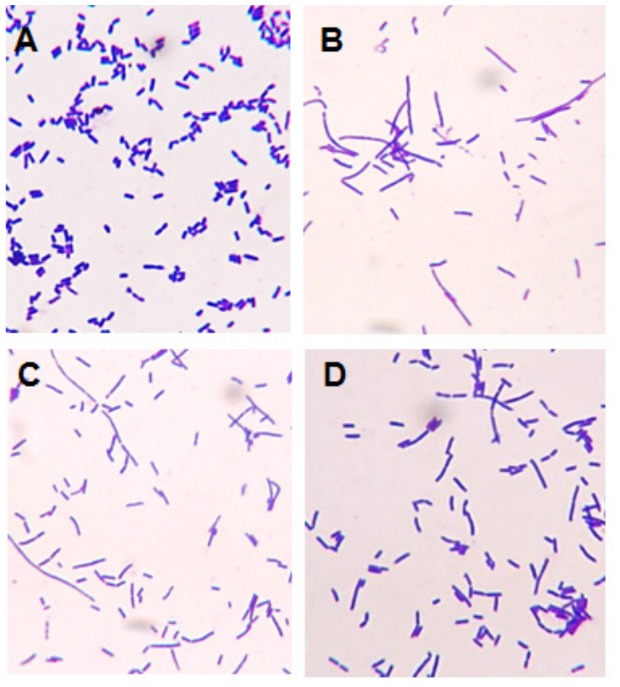
Morphology of *E. coli* cells treated with peptides. The cells were incubated with peptides for 3°C and observed using light microscopy at 1000×magnification under oil emersion. A. No peptide control; B. PuroA 32 µg/mL; C. Pina-M 8 µg/mL; D. Indolicidin 32 µg/mL.

Certain drugs which inhibit DNA synthesis in *E. coli* induce filamentation, wherein rod-shaped cells continue to grow in size but cease to divide [43]. Indolicidin, a Trp-rich AMP with broad antimicrobial activity [44], has this effect [42]; hence this test was used to determine if the peptides used in the *in vitro* DNA-binding assay could also effect *in vivo* inhibition of DNA synthesis. *E. coli* cells were treated with a range of peptide concentrations (0–250 µg/mL) for 3 h and inspected by light microscopy, using a no-peptide negative control and indolicidin (32 µg/mL) as positive control. PuroA, Pina-M, Pina-R39G, HinA and GSP-5D induced filamentation at lower concentrations (8–64 µg/mL) compared to PuroB (Figure 4; Figure S5 in File S1; Table 4), while Pina-W→F was unable to affect this phenotype. Among the PINB-based peptides, only PuroB and Hinb peptides induced filaments at 250 µg/mL. All peptides with substitutions, i.e., Pinb-B (Gly46Ser), Pinb-D (Trp44Arg), Pinb-L (Lys45Gln) and Pinb-Q (Trp44Leu) lacked this ability, agreeing with the results of the gel retardation test.

## Discussion

A number of studies have established that PIN proteins, as well as synthetic peptides designed based on their TRD, have antibacterial and/or antifungal properties, leading to the transgenic use of *Pin* genes for testing the *in vivo* biotic defence capabilities of PIN proteins and contemplations of ectopic applications of the peptides for control of human infections [7]. However, the mechanism of membrane activity by these proteins and peptides is as yet unresolved. This is a significant factor for considering their applications in agriculture, food safety or health contexts. Hence the aims of the present work were to investigate the nature of interactions of PIN-based peptides with the membranes of bacterial and fungal cells. These were investigated through a number of methods: use of membrane-mimicking vesicles, transmission and scanning electron microscopy, tests for *in vivo* yeast cell permeabilisation, and assays for *in vitro* DNA-binding and *in vivo* blockage of DNA replication.

The broad range of antimicrobial activities and low haemolytic activity of PIN-based peptides noted earlier [17]–[19] showed a high selectivity towards microbial cells. The attribute is shared by other Trp-rich AMPs including LfcinB and Tritrpticin [45], making them attractive therapeutic agents. The selectivity stems from electrostatic interactions between the CAP (mainly its Lys/Arg residues) and the anionic head-groups of bacterial membrane lipids [46]. The peptides which earlier demonstrated the highest AMP activity, PuroA, Pina-M and Pina-W→F [18], showed strong preference for the negatively charged DOPG:DOPC (microbial membrane-mimicking) vesicles over the zwitterionic DOPC (mammalian membrane-mimicking) vesicles in the present work. This is consistent with previous studies using model membranes with PuroA [17]; combi-1; (a synthetic hexapeptide RRWWRF-NH2; [47]) and synthetic CAPs rich in Lys, Phe and Trp (cationic, hydrophobic) [48]. The Trp residues of PuroA were found to bury deeper into DOPG than DOPC vesicles, and it was suggested that the cation-π interactions between Trp and an adjacent Arg (lacking in PuroB) may facilitate deeper insertion of PuroA into bacterial membranes [17]. Replacement of the Trps with Phe (Pina-W→F) decreased the membrane-perturbing ability of the TRD-based peptide towards the bacterial-mimicking membranes, while not significantly affecting the antimicrobial activity [18]. The reduced positive charge and fewer Trp residues are attributed to the lower potency of PuroB as compared to PuroA [17]. The anionic membrane charges may thus contribute to the selectivity of the basic PIN proteins [26] and peptides [17], [18] towards bacterial membranes and lack of toxicity towards murine cells [26].

Lack of electrostatic attraction may not be the only relevant factor for neutral membranes, as some CAPs including indolicidin can disrupt such membranes [44]. PuroA and Pina-M both led to a small degree of calcein leakage from the DOPC LUVs at high concentrations (125 µg/mL), Pina-M showing stronger activity (54.5%) than PuroA (46%). The enhanced affinity of Pina-M to neutral membranes may be due to its Pro35Ser substitution; computer simulations with synthetic linear Lys- and Ser-rich bioactive peptides found that the OH group of Ser can form H-bonds with membrane phospholipids [49]. Interestingly, replacement of Trp residues with Phe in the PuroA-based peptide effectively abolished leakage from the DOPC LUVs, even at high concentrations (125 µg/mL). Such observations have also been reported for Tritrpticin (Trp-rich AMP), with the lack of permeability towards mammalian cell membranes important for potential therapeutic applications [32]. Other factors that influence permeabilisation of membranes with low surface charge include hydrophobic moment, oligomerization and the specific sequence/orientation of the peptide [46]. Such factors need to be investigated for both the PIN proteins and peptides, to obtain insights into (i) why mutations at residues other than the Trps (e.g., Pro35Ser; *Pina-D1m,* Gly46Ser; *Pinb-D1b*) or outside the TRD (e.g., Leu60Pro; *Pinb-D1c*) affect the affinity of the PIN proteins for the starch granule membranes in relation to wheat grain texture [6]; and (ii) whether such mutations can also affect the antimicrobial properties of the peptides and any *in vivo* activities of the proteins in biotic defence.

Membrane-active AMPs can be divided into those that permeabilise cell membranes resulting in cell lysis, and those that permeate the membranes without cell lysis to gain access to intracellular target(s) [21]. LUVs mimicking bacteria, when treated with PuroA and Pina-M, induced dye leakage but not complete lysis after 5 min treatment, confirmed by visualisation using TEM. The non-lysis seems shared with the Trp-rich AMP indolicidin, which can permeabilise both membranes of *E. coli* but does not cause lysis [50]. Visualisation of peptide-treated negatively charged LUVs using TEM indicated changes such as rough surface, swelling, and leakage of contents. To our knowledge, this is the first report using TEM to provide direct proof of loss of membrane integrity caused by these peptides.

The unicellular fungal cells (*S. cerevisiae*) provided an ideal *in vivo* model to visualise the effect of peptides via uptake of PI, a dye for which intact membranes are normally impermeable to, since these cells also have negatively charged membranes, similar to bacteria [22]. A yeast complementation test showed that PINs can interact with the plasma membrane of *S. cerevisiae* via the TRD, with the Trp residues in PINA and Lys residues in PINB found to be the limiting factor in binding [51]. In our work, 100% of the *S. cerevisiae* cells became permeable to PI when treated with PuroA or Pina-M (both containing 5 Trp residues) at equal to, or greater than, their respective MIC values for yeast. Less permeabilisation was observed for the peptides with a decreased number of, or lack of, Trp residues (PuroB and Pina-W→F) at their MIC levels. The results thus establish that the Trp residue(s) is (are) important for membrane permeabilisation, and support the findings of Evrard et al. [51] for PuroA; however, the reduced permeation of the Lys-containing PuroB questions their findings on PINB. Differences in activity have been reported for the PIN proteins, with PINB showing more activity towards fungal pathogens than PINA [9], while the activity of both PINs against select bacterial species was the same [10]. However, the functionality of other candidate factors such as certain hydrophobic residues may also be relevant for any activities involving proteins compared to the much shorter peptides.

The peptide-treated *S. cerevisiae* cells support a non-lytic mechanism, showing intact cells with pore-like structures in the membranes. Peptide sequences and concentrations appear important for the amount of membrane penetration, as more pronounced pores were seen for peptides with a higher proportion of Trp residues and at higher peptide concentrations. Short CAPs (∼20 residues) are generally considered unlikely to form stable pores, as they require membrane-spanning α-helices, but they may form transient pores [46]. There are two leading models for disruption of bacterial membranes by short AMPs; the ‘carpet model’ [52] and the ‘aggregate model’ [21]. The ‘carpet model’ does not depend upon a particular length or sequence conformation, and proposes a four-step mechanism wherein: i) the peptide binds to the negatively charged headgroups of membrane phospholipids via its basic residues, covering it like a carpet; ii) its hydrophobic regions align with lipids in the hydrophobic core; iii) more peptides bind until a threshold is reached that induces membrane curvature and formation of lipid micelle; iv) the micelle cause formation of pores, which allow entry of materials including peptides. Glukhov et al. [46] confirmed this mechanism (the ‘grip and dip’ mechanism) for short (∼17 residue) Lys/Trp-rich AMPs. Importantly, Kooijman et al. [23] also confirmed two types of interactions for PIN proteins: electrostatic interactions between the Arg/Lys in the TRD and the phosphate headgroups of lipids, and hydrophobic interactions between the Trps and lipid tails. The alternative ‘aggregate model’ proposes formation of lipid/peptide aggregates (‘micelle-like’ complexes) that form unstable bilayer spanning channels which collapse and translocate the peptides into the cytoplasm [21]. Jing et al. [17] showed that PuroA forms a well-defined amphipathic structure in the presence of sodium dodecyl sulphate (SDS) by association of the positively charged side-chains to the polar face of the micelles, and suggested that it does not penetrate deeply to form stable channels/pores, but may perturb the membrane through inducing positive curvature. Our observations of non-lysis of yeast cells and induction of pore formation by TRD-based peptides support this theory, and are probably indicative of a ‘carpet model’ of membrane activity. Importantly, this model is proposed for the bactericidal TRD of PINs only. The protein has the potential for forming membrane spanning helices, that could form stable pores or channels, as previously reported [25], [26].

Once a non-lytic AMP crosses the membrane, a number of intracellular targets are available to it, and the interactions can result in inhibitions of DNA, RNA, protein or cell wall syntheses, effects on protein folding, enzyme activities, or actions of certain peptides [21]. The well-studied Trp-rich AMP indolicidin does not induce cell lysis, instead accessing an intracellular target (DNA synthesis apparatus) [40], [42]. Hence it was used as a ‘positive control’ for both DNA-binding related assays (gel retardation and *E. coli* filamentation) conducted here. Certain drugs which block cell division by inhibiting DNA synthesis in *E. coli* induce filamentation, including the antibiotic nalidixic acid [43], and the AMP indolicidin [42]. Filaments in bacteria result when rod-shaped cells cease to divide but continue to grow [53]. The PINA-based peptides showed strong gel retardation of plasmid DNA, and the results were complemented by the filamentation assay. The basic amino acids are likely to be responsible for nucleic acid binding. Studies with indolicidin showed that when 3 or 4 Trps were replaced with Lys, the peptides bound more strongly to DNA and increased the cellular uptake of the peptide [54]. In our study, high net positive charge for the peptides was found to be important for strong DNA binding ability. However, there were some exceptions; e.g., Pina-R39G had a net charge of +2 but a high DNA binding ability, while Pinb-D, which has a Trp to Arg replacement and a net charge to +3, did not show any notable binding. It thus appears that the number of Trp residues may also be relevant to DNA binding, Pina-R39G having five Trps but Pinb-D having only two. This observation supports the two-stage model proposed by [40] for CAPs, wherein the positively charged residues electrostatically bind to the phosphate groups of DNA, then can insert into DNA duplexes, with the Trps stacking between the sugars.

Some AMPs can bind to DNA to cause direct DNA damage, or alternatively, they can bind to the Holliday junctions (HJ; branched DNA intermediates), such as the Trp/Arg-rich hexapeptides designed by Gunderson & Segall [55] which have high affinity for HJs and cause a decrease in DNA synthesis due to interference with DNA repair and replication forks. Failure to resolve HJs induces the SOS response [53], leading to cell growth inhibition (seen as filamentation in *E. coli*) [55]. Investigations along these lines are required to further characterise the DNA-related inhibition mechanisms of the PIN peptides and proteins. Further, some AMPs contain short domains which are stand-alone ‘cell-penetrating peptides’ (CPPs), and often Arg, Trp and/or Lys rich [56]. Such CPPs are currently being developed for delivery of DNAs, oligonucleotides, proteins or other substances into cells, and some are under clinical trials [56]. It will thus be interesting to investigate whether the PuroA and PuroB peptides may contain shorter CPPs.

## Supporting Information

File S1 Figure S1, A Standard curve for phosphate (P) concentration. The standard curve was prepared using H_2_PO_4_ prepared in MilliQ water, and the absorbance measured at 820 nm. Figure S2, Calcein dye release from LUVs by PuroA and Pina-M peptides. % Release of calcein dye from dye-filled LUVs over 5 minutes of incubation with a peptide at a range of final concentrations (8–125 µg/mL). **A:** Calcein release from DOPC:DOPG (1∶3) LUVs by PuroA; **B:** Calcein release from DOPC LUVs by PuroA; **C:** Calcein release from DOPC:DOPG (1∶3) LUVs by Pina-M; **D:** Calcein release from DOPC LUVs by Pina-M; **E:** Calcein release from DOPC:DOPG (1∶3) LUVs by Pina-W→F; **F:** Calcein release from DOPC LUVs by Pina-W→F; **G:** Calcein release from DOPC:DOPG (1∶3) LUVs by PuroB; **H:** Calcein release from DOPC LUVs by PuroB Positive control (•): LUVs treated with 1.0% Triton X-100; negative control (○): untreated LUVs. Figure S3, a. Light and fluorescence microscopy of yeast cells (positive and negative controls). Untreated *S. cerevisiae* cells: **A)** Light microscopy; **B)** Fluorescence microscopy; *S. cerevisiae* cells treated with indolicidin (125 µg/mL; MIC level); **C)** Light microscopy; **D)** Fluorescence microscopy. Magnification 400×. **b.** Light and fluorescence microscopy of yeast cells treated with PuroA. *S. cerevisiae* cells treated with: PuroA 64 µg/mL: A) Light microscopy and B) Fluorescence microscopy; PuroA 125 µg/mL: C) Light microscopy and D) Fluorescence microscopy; PuroA 250 µg/mL: E) Light microscopy and F) Fluorescence microscopy; PuroA 500 µg/mL: G) Light microscopy and H) Fluorescence microscopy. Magnification 400×. MIC for PuroA against *S. cerevisiae* 125 µg/mL. **c.** Light and fluorescence microscopy of yeast cells treated with Pina-M. *S. cerevisiae* cells treated with: Pina-M 64 µg/mL: A) Light microscopy and B) Fluorescence microscopy; Pina-M 125 µg/mL: C) Light microscopy and D) Fluorescence microscopy; Pina-M 250 µg/mL: E) Light microscopy and F) Fluorescence microscopy; Pina-M 500 µg/mL: G) Light microscopy and H) Fluorescence microscopy. Magnification 400×. MIC for Pina-M against *S. cerevisiae* 125 µg/mL. **d.** Light and fluorescence microscopy of yeast cells treated with Pina-M. *S. cerevisiae* cells treated with: Pina-W→F 64 µg/mL: A) Light microscopy and B) Fluorescence microscopy; Pina- W→F 125 µg/mL: C) Light microscopy and D) Fluorescence microscopy; Pina- W→F 250 µg/mL: E) Light microscopy and F) Fluorescence microscopy; Pina- W→F 500 µg/mL: G) Light microscopy and H) Fluorescence microscopy. Magnification 400×. MIC for Pina- W→F against *S. cerevisiae* 250 µg/mL. **e.** Light and fluorescence microscopy of yeast cells treated with PuroB. *S. cerevisiae* cells treated with: PuroB 64 µg/mL: A) Light microscopy and B) Fluorescence microscopy; PuroB 125 µg/mL: C) Light microscopy and D) Fluorescence microscopy; PuroB 250 µg/mL: E) Light microscopy and F) Fluorescence microscopy; PuroB 500 µg/mL: G) Light microscopy and H) Fluorescence microscopy. Magnification 400×. MIC for PuroB against *S. cerevisiae* 250 µg/mL. Figure S4, **a.** Interaction of PuroA, Pina-M, Pina-R39G, Pina-W→F, HinA and GSP-5D with plasmid DNA. Binding of peptides to DNA assessed by measuring the retardation of plasmid DNA (100 ng; pBluescript SK+) migration through an agarose gel. The peptide concentration indicated above each lane represents 0, 16, 32, 64, 125, 250 and 500 µg/mL. **b.** Interaction of PuroB, Pinb-B, Pinb-D, Pinb-L, Pinb-Q, Hinb1, Hinb1a and indolicidin with plasmid DNA. Binding of peptides to DNA assessed by measuring the retardation of plasmid DNA (100 ng; pBluescript SK+) migration through an agarose gel. The peptide concentration indicated above each lane represents 0, 16, 32, 64, 125, 250 and 500 µg/mL. Figure S5, Morphology of *E. coli* cells treated with peptides. The cells were incubated with peptides for 3 h at 37°C and observed using light microscopy at 1000×magnification under oil emersion. **a:** Treatment with PuroA, Pina-M, Pina-R39G, Pina-W→F and HinA, and no-peptide control. **A.** PuroA 16 µg/mL; **B.** Pina-M 8 µg/mL; **C.** Pina-R39G 64 µg/mL; **D.** PinaW-F 250 µg/mL; **E.** HinA 32 µg/mL; **F.** no-peptide control. **b:** Treatment with PuroB, Pinb-B, Pinb-D, Pinb-L, Pinb-Q, Hinb1, Hinb1a, and no- peptide control. **A.** PuroB 250 µg/mL; **B.** Pinb-B 250 µg/mL; **C.** Pinb-D 250 µg/mL; **D.** Pinb-L 250 µg/mL; **E.** Pinb-Q 250 µg/mL; **F.** Hinb1 250 µg/mL; **G.** Hinb1a 250 µg/mL; **H.** no-peptide control. **c:** Treatment with GSP-5D, indolicidin, and no-peptide control. **A.** GSP-5D 64 µg/mL; **B.** Indolicidin 32 µg/mL; **C.** no peptide control. Table S1, Average phospholipid concentrations of the prepared LUVs.
(DOCX)
