# Supplementary material for: The Antimicrobial Domains of Wheat Puroindolines Are Cell-Penetrating Peptides with Possible Intracellular Mechanisms of Action
Source: PLoS One. 2013 Oct 2;8(10):e75488. doi: 10.1371/journal.pone.0075488 (PMC3788796; doi:10.1371/journal.pone.0075488)
Supplement: File S1 — Figure S1, A Standard curve for phosphate (P) concentration. The standard curve was prepared using H2PO4 prepared in MilliQ water, and the absorbance measured at 820 nm. Figure S2, Calcein dye release from LUVs by PuroA and Pina-M peptides. % Release of calcein dye from dye-filled LUVs over 5 minutes of incubation with a peptide at a range of final concentrations (8–125 µg/mL). A: Calcein release from DOPC:DOPG (1∶3) LUVs by PuroA; B: Calcein release from DOPC LUVs by PuroA; C: Calcein release from DOPC:DOPG (1∶3) LUVs by Pina-M; D: Calcein release from DOPC LUVs by Pina-M; E: Calcein release from DOPC:DOPG (1∶3) LUVs by Pina-W→F; F: Calcein release from DOPC LUVs by Pina-W→F; G: Calcein release from DOPC:DOPG (1∶3) LUVs by PuroB; H: Calcein release from DOPC LUVs by PuroB Positive control (•): LUVs treated with 1.0% Triton X-100; negative control (○): untreated LUVs. Figure S3, a. Light and fluorescence microscopy of yeast cells (positive and negative controls). Untreated S. cerevisiae cells: A) Light microscopy; B) Fluorescence microscopy; S. cerevisiae cells treated with indolicidin (125 µg/mL; MIC level); C) Light microscopy; D) Fluorescence microscopy. Magnification 400×. b. Light and fluorescence microscopy of yeast cells treated with PuroA. S. cerevisiae cells treated with: PuroA 64 µg/mL: A) Light microscopy and B) Fluorescence microscopy; PuroA 125 µg/mL: C) Light microscopy and D) Fluorescence microscopy; PuroA 250 µg/mL: E) Light microscopy and F) Fluorescence microscopy; PuroA 500 µg/mL: G) Light microscopy and H) Fluorescence microscopy. Magnification 400×. MIC for PuroA against S. cerevisiae 125 µg/mL. c. Light and fluorescence microscopy of yeast cells treated with Pina-M. S. cerevisiae cells treated with: Pina-M 64 µg/mL: A) Light microscopy and B) Fluorescence microscopy; Pina-M 125 µg/mL: C) Light microscopy and D) Fluorescence microscopy; Pina-M 250 µg/mL: E) Light microscopy and F) Fluorescence microscopy; Pina-M 500 µg/mL: G) Light microscopy an [file pone.0075488.s001.docx]

**Supplementary data**

**Fig. S1 A Standard curve for phosphate (P) concentration.**

The standard curve was prepared using H_2_PO_4_ prepared in MilliQ water, and the absorbance measured at 820 nm.

**A.**


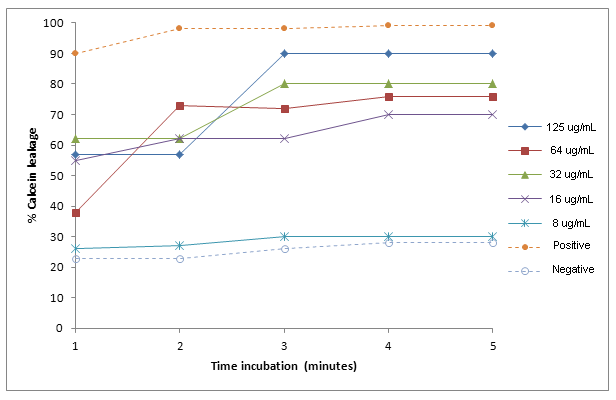


**B.**


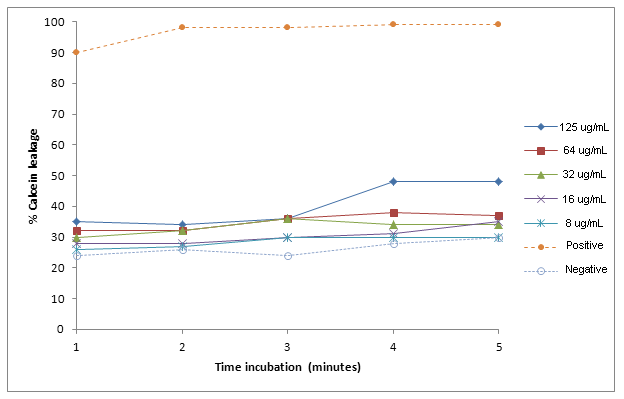


**C.**

**
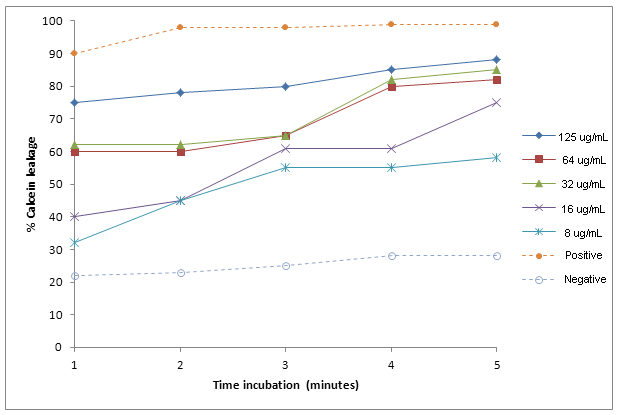
**

**D.**


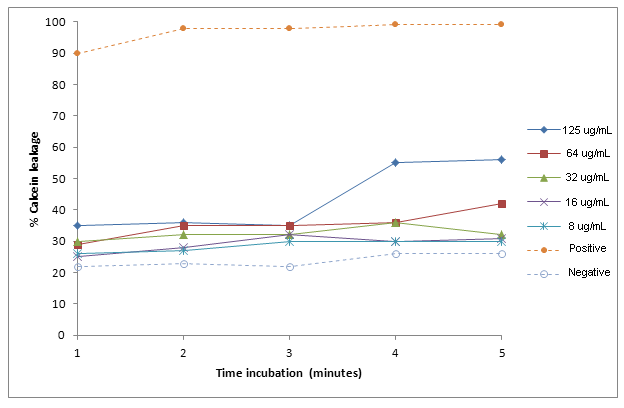


**E.**

**
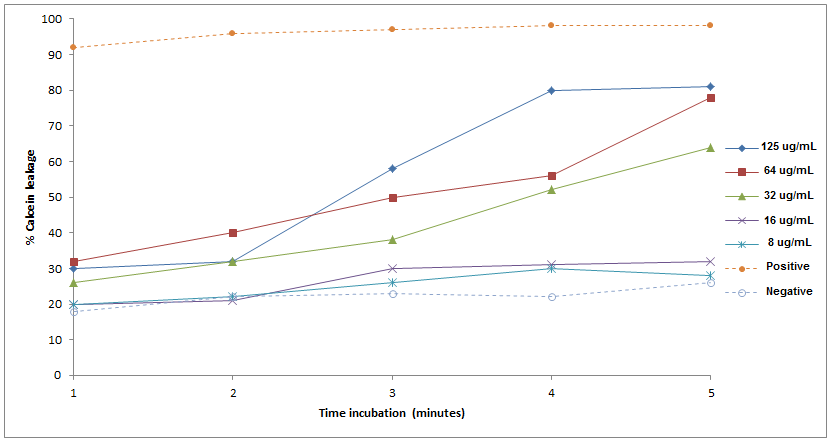
**

**F.**

**
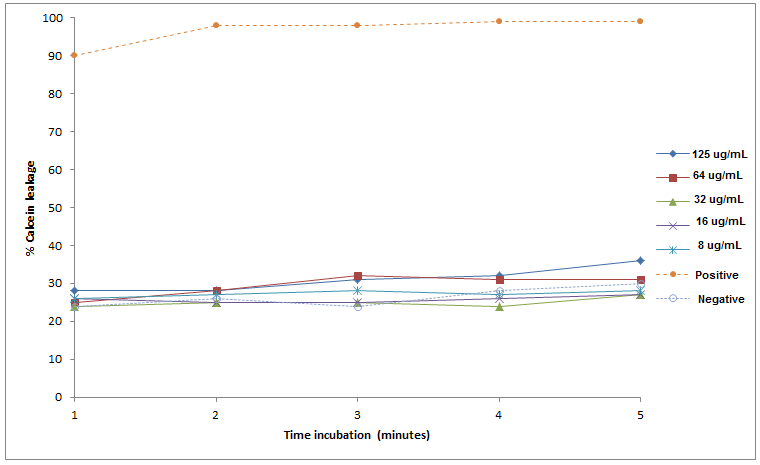
**

**G.**

**
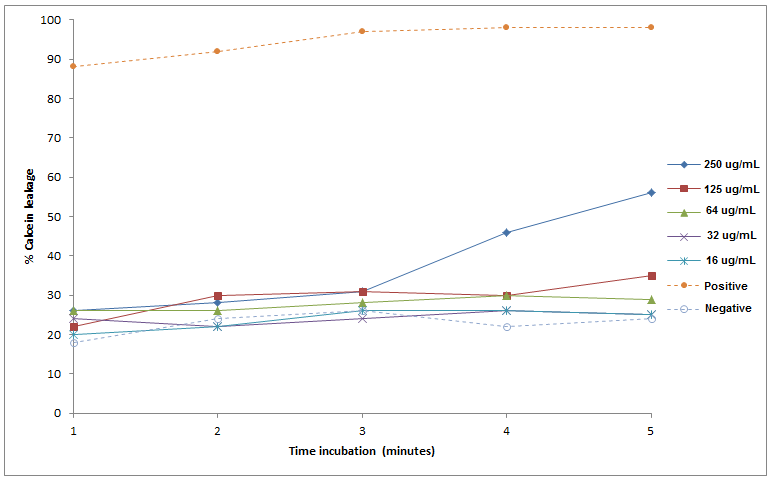
**

**H.**

**
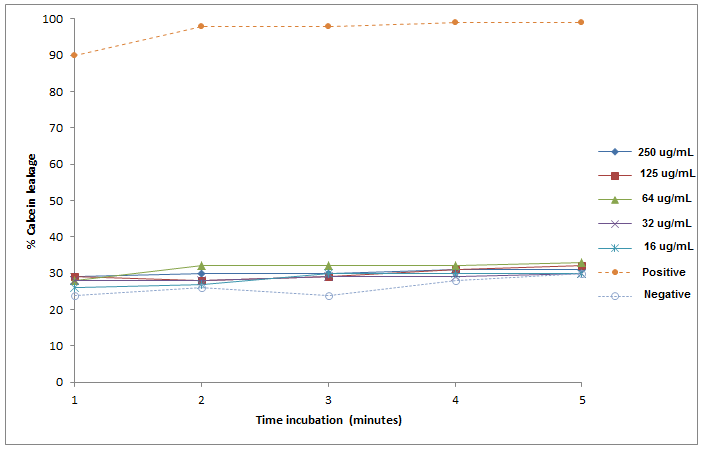
**

**Fig. S2. Calcein dye release from LUVs by PuroA and Pina-M peptides.**

% Release of calcein dye from dye-filled LUVs over 5 minutes of incubation with a peptide at a range of final concentrations (8-125 µg/mL).

A**:** Calcein release from DOPC:DOPG (1:3) LUVs by PuroA; B: Calcein release from DOPC LUVs by PuroA; C: Calcein release from DOPC:DOPG (1:3) LUVs by Pina-M; D: Calcein release from DOPC LUVs by Pina-M; E: Calcein release from DOPC:DOPG (1:3) LUVs by Pina-W⭢F; F: Calcein release from DOPC LUVs by Pina-W⭢F; G: Calcein release from DOPC:DOPG (1:3) LUVs by PuroB; H: Calcein release from DOPC LUVs by PuroB Positive control (●): LUVs treated with 1.0% Triton X-100; negative control (○): untreated LUVs.

**
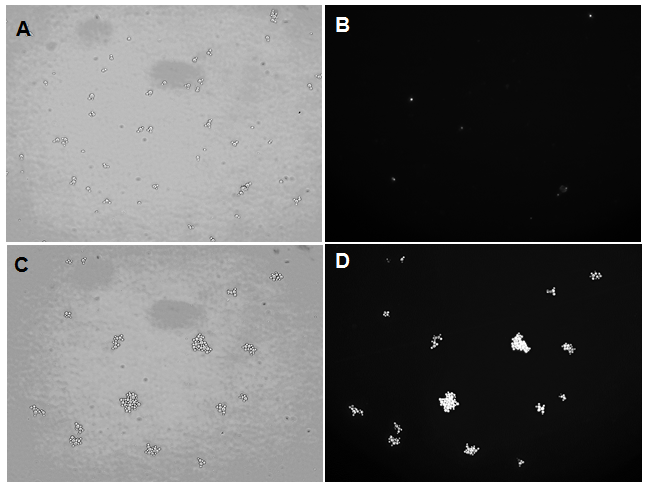
**

**Fig. S3a Light and fluorescence microscopy of yeast cells (positive and negative controls).**

Untreated *S. cerevisiae* cells: A) Light microscopy; B) Fluorescence microscopy;

*S. cerevisiae* cells treated with indolicidin (125 µg/mL; MIC level): C) Light microscopy; D) Fluorescence microscopy. Magnification 400×.

**
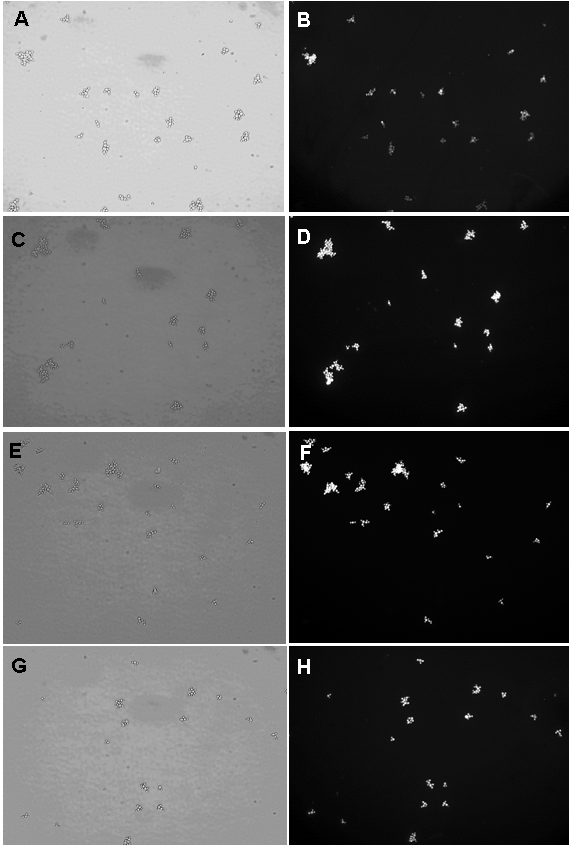
**

**Fig. S3b Light and fluorescence microscopy of yeast cells treated with PuroA.**

*S. cerevisiae* cells treated with:

PuroA 64 µg/mL: A) Light microscopy and B) Fluorescence microscopy;

PuroA 125 µg/mL: C) Light microscopy and D) Fluorescence microscopy;

PuroA 250 µg/mL: E) Light microscopy and F) Fluorescence microscopy;

PuroA 500 µg/mL: G) Light microscopy and H) Fluorescence microscopy.

Magnification 400×. MIC for PuroA against *S. cerevisiae* 125 µg/mL.

**
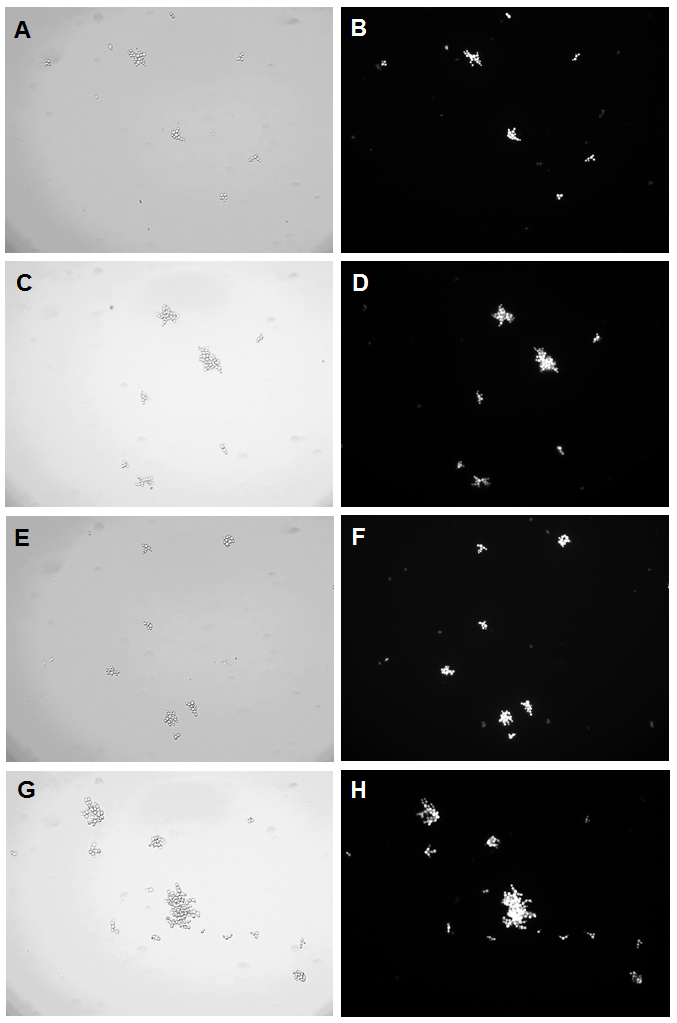
**

**Fig. S3c Light and fluorescence microscopy of yeast cells treated with Pina-M.**

*S. cerevisiae* cells treated with:

Pina-M 64 µg/mL: A) Light microscopy and B) Fluorescence microscopy;

Pina-M 125 µg/mL: C) Light microscopy and D) Fluorescence microscopy;

Pina-M 250 µg/mL: E) Light microscopy and F) Fluorescence microscopy;

Pina-M 500 µg/mL: G) Light microscopy and H) Fluorescence microscopy.

Magnification 400×. MIC for Pina-M against *S. cerevisiae* 125 µg/mL.

**
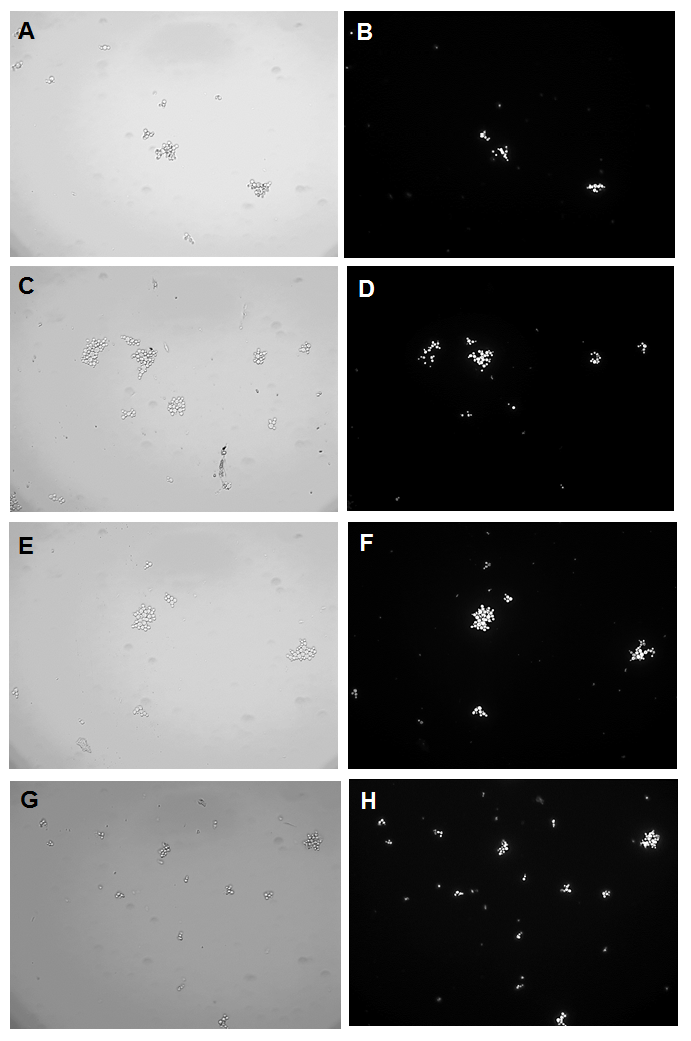
**

**Fig. S3d Light and fluorescence microscopy of yeast cells treated with Pina-M.**

*S. cerevisiae* cells treated with:

Pina-W⭢F 64 µg/mL: A) Light microscopy and B) Fluorescence microscopy;

Pina- W⭢F 125 µg/mL: C) Light microscopy and D) Fluorescence microscopy;

Pina- W⭢F 250 µg/mL: E) Light microscopy and F) Fluorescence microscopy;

Pina- W⭢F 500 µg/mL: G) Light microscopy and H) Fluorescence microscopy.

Magnification 400×. MIC for Pina- W⭢F against *S. cerevisiae* 250 µg/mL.

**
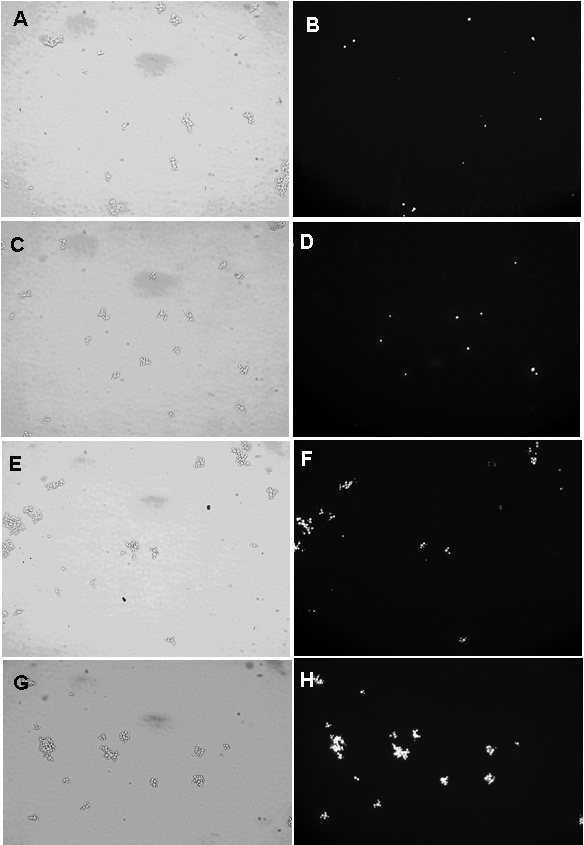
**

**Fig. S3e Light and fluorescence microscopy of yeast cells treated with PuroB.**

*S. cerevisiae* cells treated with:

PuroB 64 µg/mL: A) Light microscopy and B) Fluorescence microscopy;

PuroB 125 µg/mL: C) Light microscopy and D) Fluorescence microscopy;

PuroB 250 µg/mL: E) Light microscopy and F) Fluorescence microscopy;

PuroB 500 µg/mL: G) Light microscopy and H) Fluorescence microscopy.

Magnification 400×. MIC for PuroB against *S. cerevisiae* 250 µg/mL.

**
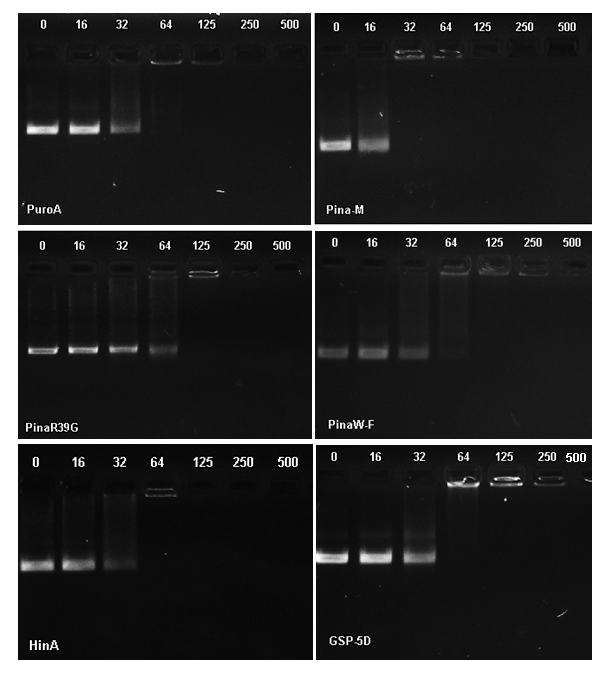
**

**Fig. S4a. Interaction of PuroA, Pina-M, Pina-R39G, Pina-W→F, HinA and GSP-5D with plasmid DNA.**

Binding of peptides to DNA assessed by measuring the retardation of plasmid DNA (100 ng; pBluescript SK+) migration through an agarose gel. The peptide concentration indicated above each lane represents 0, 16, 32, 64, 125, 250 and 500 µg/mL.

**
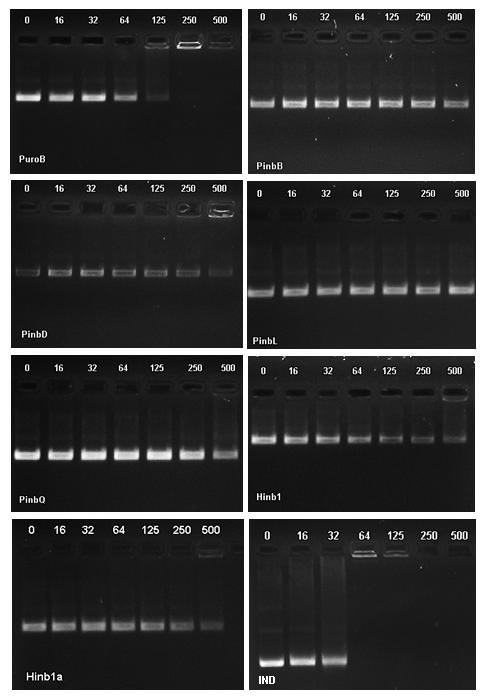
**

**Fig. S4b. Interaction of PuroB, Pinb-B, Pinb-D, Pinb-L, Pinb-Q, Hinb1, Hinb1a and indolicidin with plasmid DNA.**

Binding of peptides to DNA assessed by measuring the retardation of plasmid DNA (100 ng; pBluescript SK+) migration through an agarose gel. The peptide concentration indicated above each lane represents 0, 16, 32, 64, 125, 250 and 500 µg/mL.

**a.**

**
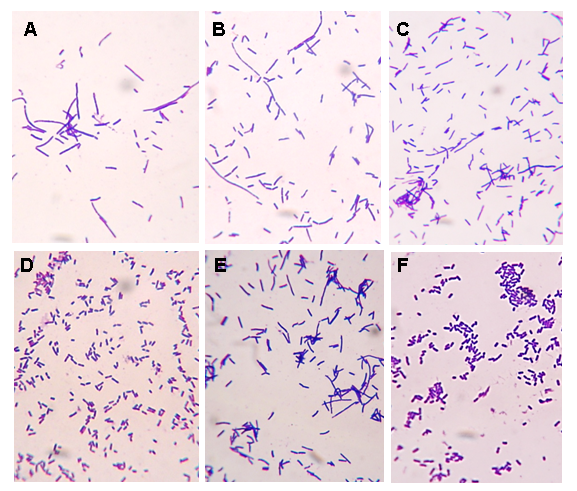
**

**b**.

**
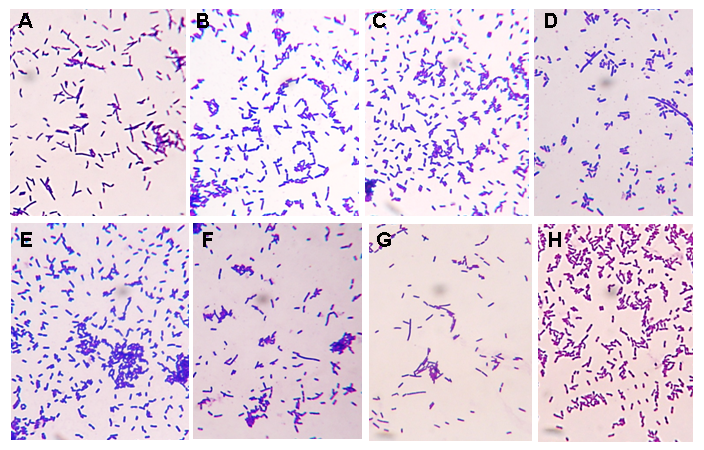
**

**c.**

**
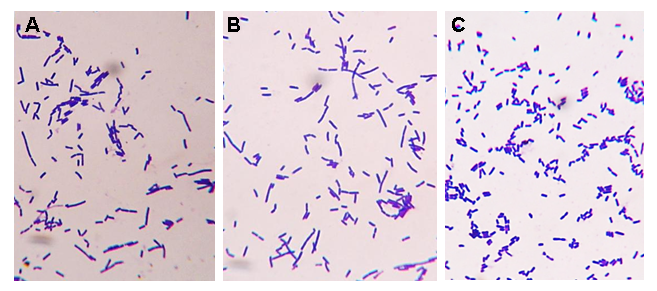
**

**Figure S5. Morphology of *E. coli* cells treated with peptides.**

The cells were incubated with peptides for 3 h at 37ºC and observed using light microscopy at 1000× magnification under oil emersion.

**a: Treatment with PuroA, Pina-M, Pina-R39G, Pina-W🡒F and HinA, and no-peptide control. A.** PuroA 16 µg/mL; **B.** Pina-M 8 µg/mL; **C**. Pina-R39G 64 µg/mL; **D**. PinaW-F 250 µg/mL; **E**. HinA 32 µg/mL; **F**. no-peptide control.

**b: Treatment with PuroB, Pinb-B, Pinb-D, Pinb-L, Pinb-Q, Hinb1, Hinb1a, and no- peptide control. A.** PuroB 250 µg/mL; **B.** Pinb-B 250 µg/mL; **C**. Pinb-D 250 µg/mL; **D**. Pinb-L 250 µg/mL; **E**. Pinb-Q 250 µg/mL; **F**. Hinb1 250 µg/mL; **G.** Hinb1a 250 µg/mL; **H.** no-peptide control.

**c: Treatment with GSP-5D, indolicidin, and no-peptide control. A:** GSP-5D 64 µg/mL; **B.** Indolicidin 32 µg/mL; **C.** no peptide control.

**Table S1. Average phospholipid concentrations of the prepared LUVs**

|  | **Absorbance 820 nm** | **Pi concentration (µg/mL)** | **Pi concentration (µM)** | **Phospholipid concentration (mM)** |
| --- | --- | --- | --- | --- |
| **DOPC:DOPG** | 1.66 | 750 | 954 | 23.85 |
| **DOPC:DOPG** | 1.52 | 690 | 877 | 21.94 |
| **DOPC** | 1.32 | 660 | 763 | 19.07 |
| **DOPC** | 1.46 | 670 | 856 | 21.24 |
